# Trends in use and impact on outcome of empiric antibiotic therapy and non-invasive ventilation in COPD patients with acute exacerbation

**DOI:** 10.1186/s13613-015-0072-x

**Published:** 2015-10-01

**Authors:** Islem Ouanes, Lamia Ouanes-Besbes, Saoussen Ben Abdallah, Fahmi Dachraoui, Fekri Abroug

**Affiliations:** Intensive Care Unit, Fattouma Bourguiba University Hospital, Rue 1er juin, 5000 Monastir, Tunisia; Laboratoire de Recherche (LR12SP15), University of Monastir, Monastir, Tunisia

**Keywords:** COPD, Acute exacerbation, ICU, Antibiotic therapy, NIV, Mortality

## Abstract

**Background:**

Empiric antibiotic therapy is routinely prescribed in patients with acute COPD exacerbations (AECOPD) requiring ventilatory support on the basis of studies including patients conventionally ventilated. Whether this practice remains valid to current management with first-line non-invasive ventilation (NIV) is unclear.

**Methods:**

In a cohort of ICU patients admitted between 2000 and 2012 for AECOPD, we analyzed the trends in empiric antibiotic therapy and in primary ventilatory support strategy, and their respective impact on patients’ outcome.

**Results:**

440 patients admitted for 552 episodes were included; primary NIV use increased from 29 to 96.7 % (*p* < 0.001), whereas NIV failure rate decreased significantly (*p* = 0.004). In parallel, ventilator-associated pneumonia (VAP) rate, VAP density and empiric antibiotic therapy use decreased (*p* = 0.037, *p* = 0.002, and *p* < 0.001, respectively). These figures were associated with a trend toward lower ICU mortality rate (*p* = 0.058). Logistic regression showed that primary NIV use per se was protective against fatal outcome [odds ratios (OR) = 0.08, 95 %CI 0.03–0.22; *p* < 0.001], whereas NIV failure, VAP occurrence, and cardiovascular comorbidities were associated with increased ICU mortality [OR = 17.6 (95 %CI 5.29–58.93), 11.5 (95 %CI 5.17–25.45), and 3 (95 %CI 1.37–6.63), respectively]. Empiric antibiotic therapy was associated with decreased VAP rate (log rank; *p* < 0.001), but had no effect on mortality (log rank; *p* = 0.793).

**Conclusions:**

The sustained increase in NIV use allowed a decrease in empiric antibiotic prescriptions in AECOPD requiring ventilatory support. Primary NIV use and its success, but not empiric antibiotic therapy, were associated with a favorable impact on patients’ outcome.

## Background

Chronic obstructive pulmonary disease (COPD) has become the third leading cause of mortality worldwide [1–3]. Its natural history is marked by the occurrence of episodes of acute exacerbations (AECOPD) contributing to a progressive decline in respiratory function and impaired quality of life [4–8]. Exacerbations are also an important source of care expenditures, as they are responsible for more than 700,000 hospitalizations annually in the USA and a total cost of $36 billion [9–12]. Intensive care unit (ICU) plays a pivotal role in the management of severe hypercapnic COPD exacerbation, where treatment relies mainly on ventilatory support, in addition to commonly administered medications and control of exacerbation triggers [13, 14].

Respiratory infections are the most frequent causes of COPD exacerbations, accounting for 50–80 % of all exacerbations, and antibiotics are commonly administered [15–19] and still recommended especially by the last GOLD guidelines, in the setting of severe COPD exacerbations [4]. This practice is based on microbiological studies showing a higher prevalence of bacteria in the airways of patients during exacerbation, in comparison with stable COPD and healthy subjects [20–22]. However, the clinical relevance of bacterial presence in the airways has been questioned, and the lack of sensitive and specific diagnosis tools adds difficulties in determining the precise proportion of bacterial infections among other causes of exacerbation to better guide antibiotic therapy [23–27]. Moreover, approximately one-third of severe exacerbation episodes remain without identified cause [22, 28–32].

A randomized controlled trial conducted between 1996 and 1999 in our ICU is frequently quoted as the main source of evidence supporting empiric antibiotic therapy in patients with AECOPD requiring ventilatory support [33]. This trial showed that the rate of in-hospital mortality in the treated group was five times lower than that recorded in the placebo group. The results also suggested that the beneficial effects of antibiotic administration might have resulted from its selective digestive decontamination-like effect, with a substantial reduction in the ventilator-associated pneumonia (VAP) rate. At the time that the study was conducted, non-invasive ventilation (NIV) was an emerging technique and failures requiring intubation were frequent (up to 80 %). Accordingly, the majority of these patients ultimately received conventional mechanical ventilation as the primary method of ventilation. NIV has since gained ground as the first-line method of ventilation in hypercapnic COPD exacerbation [34–36] and has been associated with lower complications and improved outcome and lower costs of care [37, 38]. NIV use has progressively increased in our department with a better mastering of the technique by the health-care team; in this context of changing practices, the beneficial effect of routine empiric antibiotic therapy should be reconsidered.

In this study, we describe the changing pattern of primary ventilation method in our ICU and investigate whether the increased use of NIV would impact the empirical antibiotic prescription in the exacerbation of COPD, a practice that is common and still recommended. The respective impact of the ventilation method and empiric antibiotic therapy on patient-centered outcomes is also assessed.

## Methods

This is an observational cohort study, conducted in the ten-bed medical ICU of the teaching university hospital Fattouma Bourguiba of Monastir (802 beds) during the period between 1 January 2000 and 31 December 2012. Given the observational design of the study, the institutional review board of our hospital waived the need of a formal study approval.

### Inclusion criteria

We included consecutive patients admitted to the ICU for AECOPD during the study period and requiring ventilatory support.

### Non-inclusion criteria

We did not include in the present study patients with acute respiratory failure on other chronic lung disease than COPD (asthma, pulmonary fibrosis, etc.), COPD patients with an obvious cause of acute respiratory failure (pneumonia, pneumothorax, pulmonary embolism, etc.), and patients with metastatic cancer or hematological malignancy with a poor short-term prognosis and/or with an end-of-life decision.

*Data collection* The present study is a retrospective analysis of a database with prospective collection of the following data:

#### Baseline characteristics

- Characteristics related to COPD: time course, forced expiratory volume in one second (FEV1) base (when available), regular treatments (short- or long-acting β2 agonists, inhaled or systemic corticosteroids, aminophylline, etc.), and home oxygen therapy.
- Comorbidities: diabetes, hypertension, and heart failure.

#### Characteristics of the episode of exacerbation

- Simplified Acute Physiology Score (SAPS) II [39].
- Initial arterial blood gas analysis.

#### Management

- First ventilation method used at ICU admission: conventional ventilation or NIV.
- Initial empiric antibiotic therapy or not: empiric antibiotic therapy was prescribed at the discretion of the physician in charge throughout the study period without relying on infection biomarkers such as procalcitonin or CRP.
- Other treatments used during AECOPD which was also at the physician in charge’s discretion: inhaled β2 agonists and systemic corticosteroids.
- NIV failure
- Total duration of mechanical ventilation.

#### Outcomes

- Occurrence of complications such as ventilator-associated pneumonia (VAP) and the day of onset.
- ICU mortality.
- Transfer to another ward or discharge home.
- For survivors at the end of ICU stay: the last arterial blood gas analysis before ICU discharge (when available).

### Definitions

- *COPD, COPD exacerbation, and respiratory failure* were defined according to the global initiative for chronic obstructive lung disease (GOLD) [4].
  - COPD is defined as a chronic disease whose pulmonary component is characterized by airflow limitation that is not fully reversible. Airflow limitation was deemed to be present if the post-bronchodilator ratio of forced expiratory volume in 1 s (FEV1)/forced vital capacity (FVC) ratio was <0.7. Patients with suspected COPD and without previous documentation of the FEV1/FVC ratio had pulmonary function tests routinely checked on discharge from the ICU.
  - COPD exacerbation corresponds to a change in patient’s baseline dyspnea, cough, and/or sputum requiring a change in regular medication.
  - Severe exacerbation requiring ICU admission was defined by an actual or impending acute respiratory failure, with severe hypoxemia (arterial oxygen tension, PaO2 <60 mmHg and/or arterial oxygen saturation <90 % on room air) associated with hypercapnia (arterial carbon dioxide tension (PaCO2) ≥45 mmHg and pH ≤7.35) and clinical signs of excessive respiratory muscle activity (contraction of accessory respiratory muscles and respiration rate ≥25 breaths min^−1^), and/or other organ dysfunction (shock or hemodynamic instability, neurological disorders).
- *Initial ventilation method used at ICU admission* was preferably non-invasive ventilation (NIV) in non-already intubated patients who were free from hemodynamic instability or neurological disorders.
- *NIV failure* was defined either by secondary intubation regardless of the initial NIV duration, or death during NIV. Patients were usually considered to need tracheal intubation if any of the following major criteria were present: hypercapnia with respiratory acidosis (pH ≤7.20 and below its value at inclusion); hypercapnic coma (Glasgow Coma Scale ≤8 and PaCO2 ≥60 mmHg); PaO2 <45 mmHg despite a maximum tolerated inspiratory oxygen fraction; and/or cardiac arrest.
- *Initial empiric antibiotic therapy* corresponds to that administered during the first 24 h of ICU admission for the treatment of the cause of exacerbation.
- VAP in intubated patients was diagnosed on the basis of the association of clinical criteria and quantitative culture of tracheal aspirate. In non-intubated patients, sputum culture was performed and the quality of sampling checked (quality criteria were: >25 neutrophils and <5 epithelial cells per field). VAP density was calculated for patients either under conventional or non-invasive ventilation (expressed in 1000 patient-days of ventilation).

### Statistical analysis

Data were expressed as median (25–75 percentiles interquartile ranges, IQR) and compared with the Mann–Whitney test for continuous variables; dichotomous variables were expressed with percentages and compared with the Chi-square test. Patients’ characteristics (age, gender, comorbidities, mortality, etc.) were analyzed from the index hospital admission (last dated admission), while the variables related to episodes (severity scores, pH, ventilatory modalities, empiric antibiotic therapy, VAP rate, VAP density, etc.) were analyzed for all hospitalization episodes. The analysis of trends over years was performed using Chi-square test for trend for categorical variables and Spearman’s correlation coefficient test for continuous variables.

Univariate and multivariate regression (including variables with *p* value <0.2) analyses were performed to identify risk factors associated with mortality (440 patients were analyzed, and for patients admitted several times the last episode was considered).

A value of *p* < 0.05 was considered to be statistically significant. SPSS (version 17; SPSS, Chicago, IL, USA) was used for statistical analyses.

## Results

**Fig. 1:**
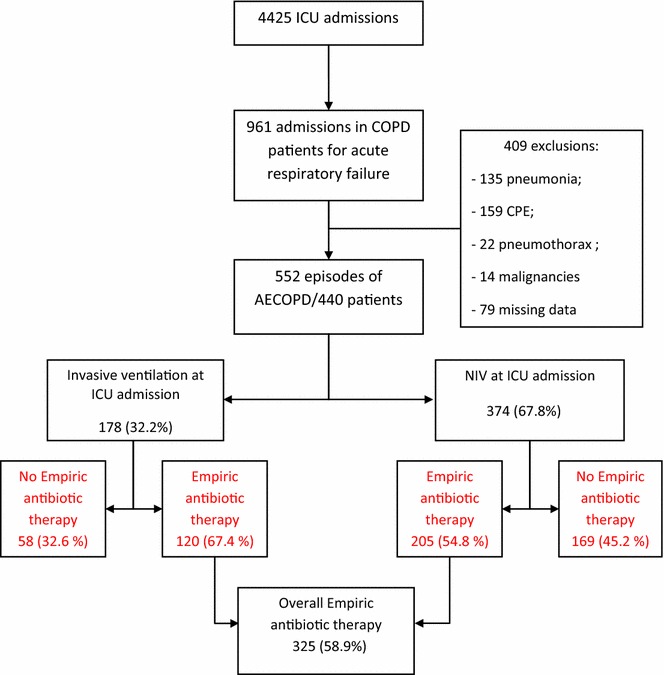
Study flowchart

During the study period, 961 out of 4425 patients admitted to the ICU had acute on chronic respiratory failure; 409 did not fulfill the definition of COPD exacerbation (principally because of an obvious reversible cause) were not included in the analysis. Of the remaining patients with AECOPD, 440 were admitted 552 times (89 were hospitalized two or more times for AECOPD) and were included in the analysis (Fig. 1). Two-thirds of patients were admitted from the emergency department. Table 1 shows the baseline characteristics of the included patients, and Table 2 reports the variables related to COPD exacerbation episodes.

**Table 1:** Baseline characteristics of the study patients

Variables	All patients: *n* = 440
Age (*n* = 440) med (IQR)	68 (61–74)
Gender (M/F)	372/68
FEV_1_ (ml) (*n* = 92) med (IQR)	965 (700–1060)
Time course of COPD (years) (*n* = 243) med (IQR)	7 (4–15)
Comorbidities	
Diabetes *n* (%)	81 (18.4)
Hypertension *n* (%)	127 (28.9)
Cardiac failure *n* (%)	36 (8.2)
Oxygen home therapy *n* (%)	93 (21.1)
Baseline treatment	
Aminophylline *n* (%)	107 (24.3)
Short-action duration ß2 mimetics *n* (%)	197 (44.7)
Long-action duration ß2 mimetics *n* (%)	46 (10.4)
Anticholinergics *n* (%)	39 (8.9)
Inhaled corticosteroids *n* (%)	116 (26.4)
Systemic corticosteroids *n* (%)	37 (8.4)

*FEV*_*1*_ forced expiratory volume in 1 s, *COPD* chronic obstructive pulmonary disease, *med* median, *IQR* inter-quartile range, *M* male, *F* female

**Table 2:** Variables related to COPD exacerbation

Variables	All hospitalisations: *n* = 552
SAPS II (*n* = 533) med (IQR)	27 (21–34)
pH at ICU admission (n = 534) med (IQR)	7.28 (7.23–7.32)
PaO2 (mmHg) (n = 534)med (IQR)	66.9 (51–90)
PaCO2 (mmHg) (*n* = 534) med (IQR)	65.9 (55.6–76.4)
HCO_3_^−^ (mmol/l) (*n* = 534) med (IQR)	31 (27.1–34.8)
CRP (mg/L) (*n* = 223) med (IQR)	41 (11.3–96.3)
WBC count (cells/µl) (*n* = 338) med (IQR)	10,800 (8000–14,200)
Modality of ventilation at ICU admission	
Invasive ventilation *n* (%)	178 (32.2)
NIV *n* (%)	374 (67.8)
NIV failure *n* (%)	63 (16.8)
Empiric antibiotic therapy at ICU admission *n* (%)	325 (58.9 %)
Bronchodilators *n* (%)	483 (87.5)
Systemic corticosteroids for exacerbation *n* (%)	199 (36.1)
VAP *n* (%)	63 (11.4)
Duration of ICU stay (days) (*n* = 552) med (IQR)	9 (6–14)

*SAPS* Simplified Acute Physiology Score, *ICU* intensive care unit, *PaO*_*2*_ arterial oxygen tension, *PaCO*_*2*_ arterial carbon dioxide tension, *HCO*_*3*_^*−*^ bicarbonate concentration, *CRP* C reactive protein, *WBC* white blood cells, *NIV* non-invasive ventilation, *VAP* ventilator associated pneumonia, *med* median, *IQR* inter-quartile range

Most patients (84.5 %) were male; their median age was 68 years (IQR 61–74) and median SAPS II score 27 (IQR 21–34). The median time course of COPD was 7 years (IQR 4–15) and the median forced expiratory volume in 1 s (FEV1) was 965 ml (IQR 700–1060).

### Changes in clinical characteristics, management, and outcomes during the study period

Table 3 summarizes the changes over the study period of the following variables: age, initial clinical severity (reflected by SAPS II score and pH at admission), the frequency of NIV use as the primary ventilation method and that of empiric antibiotic administration, the rate of VAP occurrence, and the ICU mortality rate.

**Table 3:** Changes in clinical severity, management, and outcome (552 episodes of AECOPD between 2000 and 2012)

	2000 (*n* = 31)	2001 (*n* = 81)	2002 (*n* = 56)	2003 (*n* = 56)	2004 (*n* = 40)	2005 (*n* = 29)	2006 (*n* = 33)	2007 (*n* = 37)	2008 (*n* = 49)	2009 (*n* = 61)	2010 (*n* = 19)	2011 (*n* = 30)	2012 (*n* = 30)	p*
Age, years, med (IQR)	69 (67–74)	66 (61.2–74)	70 (61–72.5)	70 (63–76.7)	69 (60–74)	65 (60.5–75)	62 (53.7–68.5)	72 (65–78)	69.5 (62–75.7)	69 (62–75)	64 (50–71.5)	62.5 (56.7–70.7)	65.5 (57.2–70.7)	0.078
SAPS II med (IQR)	28 (21–43.5)	28 (22–39.5)	25.5 (21–32)	27.5 (22–34)	29.5 (21–35)	24 (19–33)	26 (19–30)	30 (23–35)	29 (24–32)	28 (18–35)	26 (19–31.5)	22 (18–25)	27 (21.5–33)	0.018
pH at ICU admission med (IQR)	7.23 (7.15–7.28)	7.26 (7.19–7.30)	7.26 (7.20–7.30)	7.26 (7.17–7.29)	7.27 (7.22–7.30)	7.30 (7.27–7.32)	7.27 (7.21–7.32)	7.29 (7.24–7.34)	7.30 (7.28–7.33)	7.29 (7.27–7.33)	7.31 (7.26–7.35)	7.30 (7.26–7.34)	7.30 (7.25–7.34)	<0.001
Primary ventilation mode														
Intubation (%)	71	54.3	44.6	46.4	45	27.6	18.2	13.5	14.3	16.4	15.8	10	3.3	<0.001
NIV (%)	29	45.7	55.4	53.6	55	72.4	81.8	86.5	85.7	83.6	84.2	90	96.7	<0.001
NIV failure (%)	22.2	21.6	25.8	30	22.7	19	18.5	12.5	9.5	11.8	12.5	7.5	13.8	0.004
Empiric antibiotic therapy at ICU admission (%)	67.7	75.3	78.6	80.4	65	69	66.7	83.8	49	15	36.8	26.7	33.3	<0.001
VAP rate (%)	19.4	16	12.5	12.5	10	10.3	12.1	8.1	8.2	8.2	10.5	6.7	10	0.037
Global duration of ventilation, days, med (IQR)	7 (5–11)	5 (3–9)	4 (3–6)	5 (3–8)	8.5 (5–11.5)	10 (6–16)	9 (6–16)	9 (6–10)	6 (4–14)	8 (5–10)	10 (5.5–14.5)	7 (5–9)	7.5 (5–13)	<0.001
VAP Density (for 1000 patient—days of ventilation)	22.98	20.63	24.47	18.04	9.13	7.89	10.17	9.14	8.08	7.83	8.54	9.66	10.27	0.002
ICU mortality (%)	12.9	16	5.4	23.2	20	6.9	12.1	16.2	14.3	4.9	15.8	3.3	6.7	0.058

*SAPS* Simplified Acute Physiology Score, *ICU* intensive care unit, *NIV* non-invasive ventilation, *VAP* ventilator associated pneumonia, *med* median, *IQR* inter-quartile range

* Changes over time were analyzed with Chi-square test for trend (categorical variables) and Spearman’s correlation coefficient test (continuous variables)

The median age of patients did not change over the study period. However, there was a statistically significant increase in arterial pH at ICU admission (median pH varied from 7.23 in 2000 to 7.30 in 2012, *p* < 0.001). There was also a slight, but statistically significant trend toward a decrease in SAPSII score over the study period (*p* = 0.018).

**Fig. 2:**
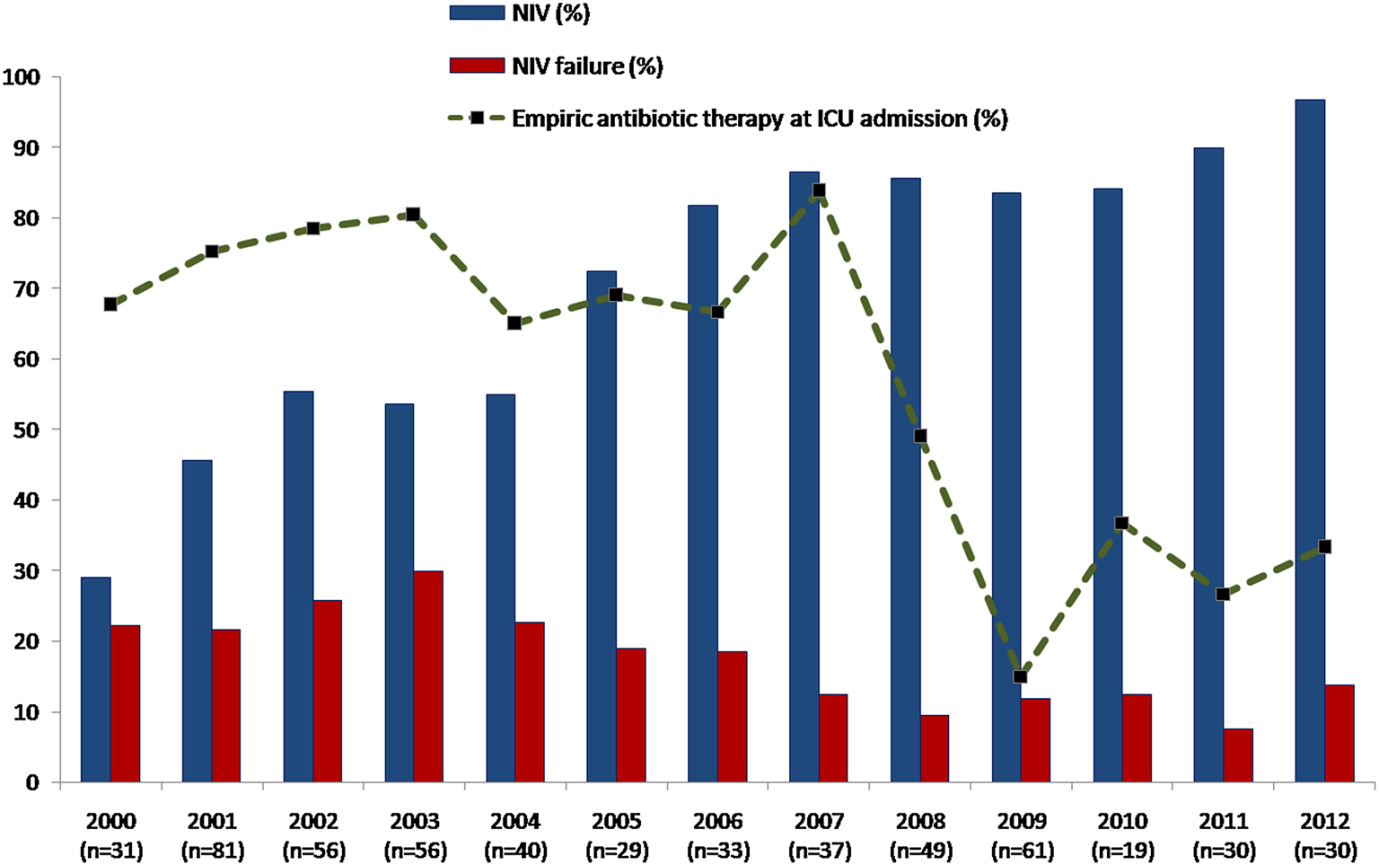
Evolution of NIV, NIV failure, and empiric antibiotic therapy use

The use of non-invasive ventilation as the primary ventilation method increased substantially from 29 % in 2000 to 96.7 % in 2012 (*p* < 0.001), and the NIV failure rate went down from around 30 % in the early 2000s to 10 % by the end of the study period (*p* = 0.004) (Table 3; Fig. 2). Meanwhile, empiric antibiotic administration was less common at the end of the study period (*p* < 0.001) (Table 3; Fig. 2). The most frequently administered antibiotics were cotrimoxazole (48 %), fluoroquinolones (37 %), amoxicillin–clavulanic acid (7 %), and a combination of third-generation cephalosporin and fluoroquinolone (5 %).

VAP density significantly decreased during the study period (22.98 in 2000 to 10.27 for 1000 patient-day of ventilation in 2012; *p* = 0.002), with a trend toward a reduced mortality rate despite wide annual variations (12.9 % in 2000 and 6.7 % in 2012, *p* = 0.058) (Table 3), an overall ICU mortality at 15.7 % (69 deaths), and a standardized mortality ratio (SMR) of 1.9 according to the SAPS II score.

### Empiric antibiotic therapy and outcomes

**Fig. 3:**
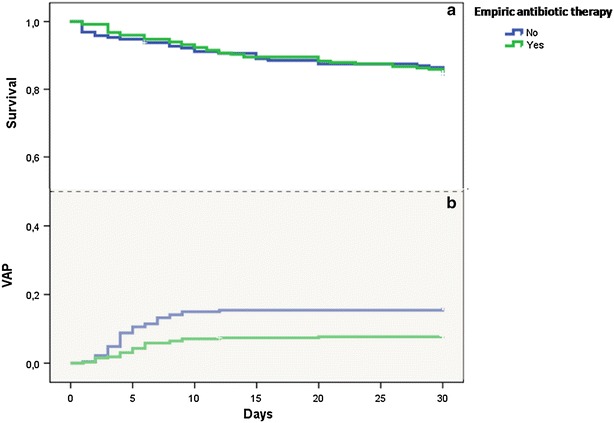
Impact of empiric antibiotic therapy on VAP and ICU mortality: analysis with Kaplan–Meier survival method shows that empiric antibiotic therapy was associated with a decrease in VAP occurrence (**b**) (log rank test, *p* < 0.001), but had no effect on mortality (**a**) (log rank test, *p* = 0.793)

Overall, empiric antibiotic therapy was administrated in 325 out of 552 AECOPD (58.9 %). The multivariate analysis showed that the empiric antibiotic therapy had no impact on the ICU mortality rate which occurred in 15.3 % of patients who received empiric antibiotics versus 16.1 % in those who did not (Chi square *p* = 0.895 and 0.793 by log rank for the Kaplan–Meier analysis; Fig. 3). Conversely, VAP was diagnosed more frequently in the group of patients who did not receive antibiotics at admission (16.3 %) than in those who received antibiotics (8 %, Chi square *p* = 0.004, <0.0001 by log rank test analysis) (Fig. 3).

### NIV and outcomes

**Fig. 4:**
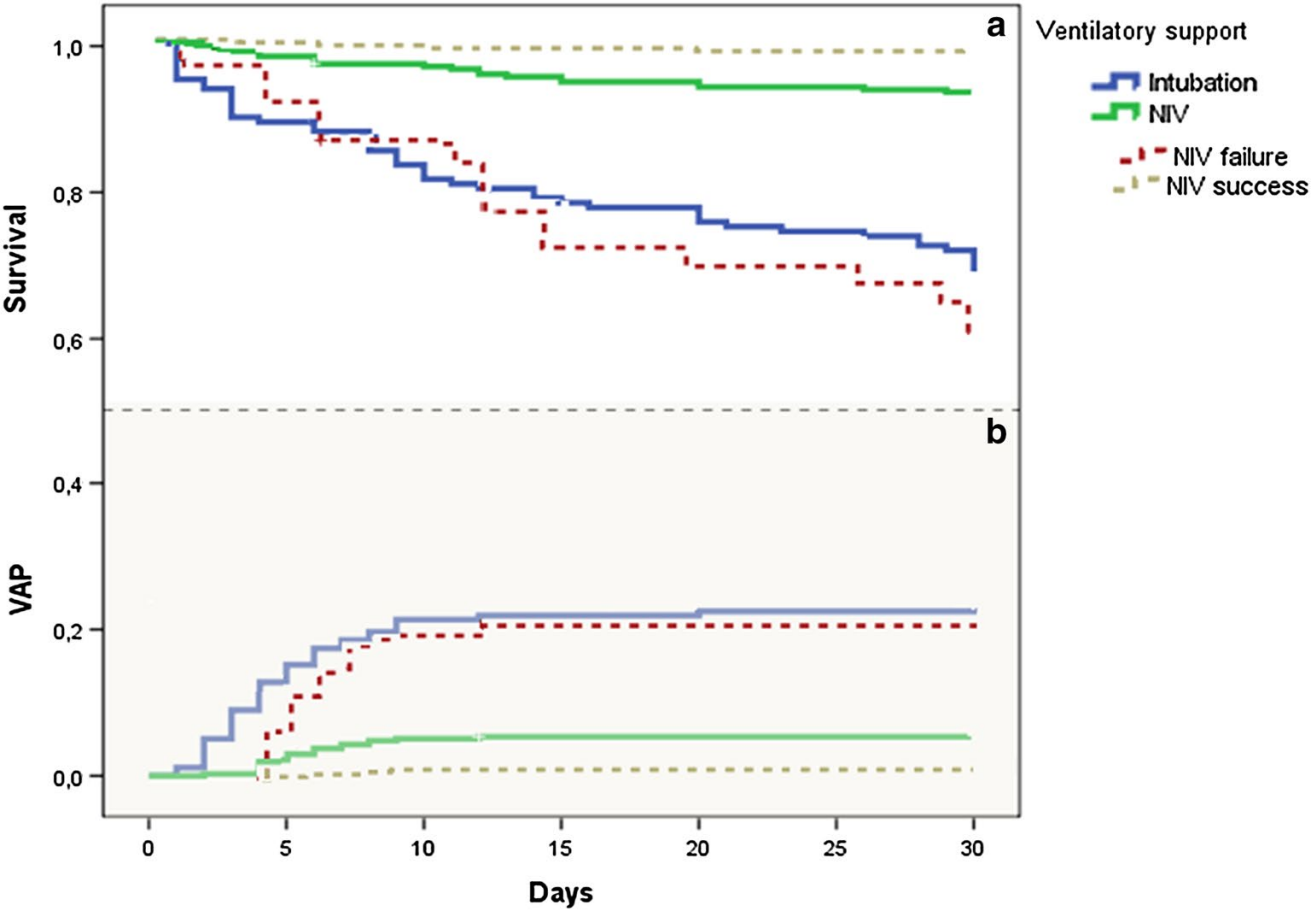
Impact of ventilation method at ICU admission and that of NIV failure on VAP rate and ICU mortality: analysis with Kaplan–Meier method shows that NIV was significantly associated with a decrease in VAP (**b**) and ICU mortality rates (**a**), (log rank test, *p* < 0.001). Conversely, NIV failure was associated with higher rates of VAP and death in the ICU compared with patients ventilated with NIV only (log rank test, *p* < 0.001) and similarly to primary invasive mechanical ventilation

At ICU admission, 374 patients (67.8 %) were started with NIV as the primary ventilation method, and 178 (32.2 %) were invasively ventilated. Both VAP and ICU mortality rates were significantly higher in patients who received invasive ventilation compared to those who were initially non-invasively ventilated with, respectively, 23.6 versus 5.6 % for VAP rate (Chi square *p* < 0.001; log rank test, *p* < 0.001) and 31.8 versus 7 % for ICU mortality (Chi square *p* < 0.001; log rank test, *p* < 0.001) (Fig. 4).

### NIV failure and outcomes

Overall, NIV as the primary ventilation method failed in 63 out of the 374 patients (16.8 %). NIV failure was associated with a significant increase in the VAP rate compared to non-failing NIV (20.6 versus 2.6 %, respectively, Chi square *p* < 0.001, log rank test, *p* < 0.001), and that of ICU mortality (41 versus 1.6 %, respectively, Chi square *p* < 0.001, log rank test, *p* < 0.001). Patients who eventually failed primary NIV had rates of VAP and mortality similar to those who received invasive ventilation as the primary ventilation method: 20.6 versus 23.6 %, and 41 versus 32 % for VAP and ICU mortality, respectively (Fig. 4).

### Factors associated with outcomes

Univariate analysis disclosed age, cardiovascular comorbidities, SAPS II score, pH at admission, NIV use as a first-line ventilation method, NIV failure, and VAP occurrence as variables associated with ICU mortality (Table 4). Multivariate logistic regression disclosed a unique protective variable against fatal outcome: the use of NIV as the primary ventilation method (odds ratio, OR = 0.08; 95 % confidence interval 0.03–0.22; *p* < 0.001). NIV failure, VAP occurrence, and cardiovascular comorbidities increased ICU mortality with respective ORs of 17.6 (95 % CI 5.29–58.93; *p* < 0.001), 11.5 (95 % CI 5.17–25.45; *p* < 0.001), and 3 (95 % CI 1.37–6.63; *p* = 0.006) (Table 5).

**Table 4:** Univariate analysis and variables associated with ICU mortality

Variables	Survivors (*n* = 372)	Dead (*n* = 68)	*p*
Age med (IQR)	68 (60–73)	70 (62–76)	*0.031*
Gender (M/F)	313/58	59/10	1.000
Time course of COPD (years) med (IQR)	7 (4–15)	8.5 (3.5–15)	0.506
FEV1 (ml) med (IQR)	860 (700–1070)	900 (775–1030)	0.489
Diabetes *n* (%)	69 (18.6)	12 (17.4)	1.000
Cardiovascular comorbidities *n* (%)	107 (28.8)	27 (39.1)	*0.116*
Oxygen home therapy *n* (%)	77 (34)	16 (29.3)	0.495
SAPS II med (IQR)	27 (21–34)	32 (26–37)	*0.003*
pH at admission med (IQR)	7.29 (7.24–7.33)	7.25 (7.19–7.30)	*0.001*
NIV at admission *n* (%)	266 (71.7)	20 (29)	*<0.001*
Invasive ventilation at admission *n* (%)	105 (28.3)	49 (71)	*<0.001*
NIV failure *n* (%)	24 (9)	16 (80)	*<0.001*
Empiric antibiotic therapy at ICU admission *n* (%)	210 (56.6)	38 (55.1)	0.895
Bronchodilators *n* (%)	204 (79.7)	37 (78.7)	0.846
Systemic corticosteroids *n* (%)	121 (33.2)	30 (44.1)	*0.097*
VAP *n* (%)	23 (6.1)	38 (55.1)	*<0.001*

*COPD* chronic obstructive pulmonary disease, *FEV*_*1*_ forced expiratory volume in 1 s, *SAPS* Simplified Acute Physiology Score, *NIV* non-invasive ventilation, *VAP* ventilator associated pneumonia, *med* median, *IQR* inter-quartile range

Variables with *p* value < 0.2 are presented in italic

**Table 5:** Multivariate analysis: independent factors associated with ICU mortality

	OR	CI 95 %		*p*
		Min	Max	
Age (per year)	0.967	0.933	1.002	0.062
SAPSII (per point)	1.017	0.981	1.054	0.371
Cardiovascular comorbidities	3.022	1.377	6.631	0.006
pH admission (per 0.01 decrease)	1.024	0.986	1.064	0.211
VAP	11.471	5.170	25.452	<0.001
NIV at admission	0.079	0.028	0.221	<0.001
NIV failure	17.663	5.294	58.932	<0.001
Systemic corticosteroids	1.543	0.752	3.166	0.237
Empiric antibiotic therapy at ICU admission	1.281	0.607	2.704	0.515

*SAPS* Simplified Acute Physiology Score, *VAP* ventilator-associated pneumonia, *NIV* non-invasive ventilation, *OR* odds ratio, *CI* confidence interval

## Discussion

Along this 13-year study, there was a sustained increase in NIV use and mastering of the intervention as reflected by a progressive decrease in the rate of failing NIV episodes in patients admitted for hypercapnic COPD exacerbation. Together with a lower use of tracheal intubation and conventional mechanical ventilation, there was a significant decrease in the rate of ventilator-associated pneumonia and a trend toward a lower ICU mortality. Changes in the ventilatory management pattern were also associated with a reduction in the systematic administration of empiric antibiotic therapy at admission which was more than halved between 2000 and 2012. ICU mortality was significantly impacted by three independent variables closely related to patients’ management (NIV use, NIV failure rate, and VAP density) and only one non-modifiable factor which was related to the patient’s previous clinical status (cardiovascular comorbidities).

The abrupt drop of empiric antibiotic therapy by the year 2007 is not related to a change in antibiotic policy. Until then, empiric antibiotic therapy was generously prescribed by physicians in charge according to evidence generated in their own ICU [33] and to recommendations [4], However, the sustained decrease in the VAP density and more generally that of the infectious burden (which paralleled the increase in NIV use) seems to have had an impact on antibiotic-prescribing patterns which relied less frequently on the almost systematic administration of empiric antibiotic therapy. The impact of the new ventilation modalities on patterns of antibiotic prescription seems to have been only postponed in comparison to the time course of the infectious risk.

The observational design of our study may have not sufficiently controlled for confounding factors impacting the choice of ventilation mode or the decision of antibiotic prescribing. However, the fact that the same physicians took these decisions throughout the study period has probably contributed to lessening the risk of selection bias. Notwithstanding, in the present study, the choice of a hard outcome such as ICU mortality precludes from other threats to observational studies such as detection bias.

Recent surveys show that NIV use has increased worldwide to become the mainstay for hypercapnic COPD exacerbation requiring ventilatory support [35, 40–43]. Several controlled studies have indeed provided compelling evidence on the effectiveness of NIV in hypercapnic exacerbation of COPD [44–47]. NIV is indeed associated with a substantial decrease in the use of intubation and invasive ventilation, the frequency of complications and adverse events, lengths of ICU and hospital stay, and mortality rates [48]. In keeping with our observation, Girou et al. [37] reported that the increase in NIV use in ICU patients admitted for COPD exacerbation (from 45 % to almost 90 % between 1994 and 2001) was associated with a lower incidence of nosocomial infections overall and specifically that of VAP [38]. NIV was also an independent factor of mortality reduction. The current study confirms previous findings on the association of NIV with a positive clinical outcome in several respects (VAP, length of stay) and extends the scope of benefits to a more sparing use of antibiotics (with potential reduction of the selection pressure and emergence of multidrug-resistant bacteria).

Despite the widespread use of antibiotics in COPD exacerbation, evidence supporting their use stems from small-sized studies [29, 49, 50]. In addition, the expected effect of antibiotics on mortality reported in meta-analyses is heavily impacted by our previous study [33] showing a substantial reduction in mortality among COPD patients with exacerbation treated with antibiotics [14, 51–53]. Furthermore, the most recent meta-analysis provided inconsistent results regarding inpatients (COPD with severe exacerbations) and outpatients with mild to moderate exacerbations [51].

The current study confirms our previous findings on the benefit of empiric antibiotic therapy regarding end points such as a shorter duration of mechanical ventilation and the length of hospital stay [33]. However, although both studies dealt with the same type of patients, cared for in a tertiary ICU by essentially the same doctors, with a similar clinical approach, the current study does not confirm the beneficial effect of empiric antibiotic therapy on mortality. Both reports differ in fact in one major aspect: the method of ventilation. In the study by Nouira et al. [33], no less than 84 % patients were eventually ventilated with conventional invasive ventilation (31 % as the primary ventilation method, and the remaining were secondarily intubated following NIV failure). The proportion of patients ventilated invasively is currently as low as 5–10 % with a lower failure rate (around 10 %) in relation with a better mastery of NIV, the so-called learning curve, as underscored by Dres et al. [54]. The use of NIV and its success appear to impact heavily patients’ outcome. As a matter of fact, our current study and the one that proceeded should not be regarded as inconsistent, and the results reported herein actually reinforce our earlier hypothesis, attributing the benefits of routine empiric antibiotic therapy to a preventive effect on the occurrence of VAP. The higher rate of intubated patients in the study by Nouira et al. [33] means also a greater risk of developing VAP compared to current patients, whereas using NIV translates into a decreased risk of VAP, which was disclosed as an independent risk factor for mortality in the current study. This assertion seems reinforced by our findings of similar outcomes (VAP and mortality rates) in patients who failed NIV and in those managed with conventional ventilation only. These patients behave worse than those with successful NIV.

Taken together, our previous publication and the current report strongly suggest that routine administration of antibiotics is probably legitimate in case of high risk of VAP (i.e., with conventional ventilation), whereas it is less justified when most COPD patients with exacerbation requiring ventilator support are managed using non-invasive ventilation, which portends a lower risk of VAP.

## Conclusion

This study shows that the increasing use of NIV in COPD exacerbation requiring ventilatory support over the last 13 years was associated with lower rates of NIV failure, VAP, and a trend toward a reduced mortality in the ICU. Changes in the first-line ventilation methods were associated with a substantial reduction in empiric antibiotic therapy prescribing. Moreover, ICU mortality was impacted by one non-modifiable factor (cardiovascular comorbidities) and three variables related to patterns of ventilatory management: the rate of NIV use as a first-line ventilation method, the rate of NIV failure, and the rate of VAP, whereas empiric antibiotic therapy had no impact on patients’ outcome.

## Abbreviations

NIV: non-invasive ventilation
COPD: chronic pulmonary obstructive disease
AECOPD: acute exacerbation of chronic pulmonary obstructive disease
VAP: ventilator-associated pneumonia
ICU: intensive care unit
SDD: selective digestive decontamination
FEV1: forced expiratory volume in 1 second
SAPS: simplified acute physiology score
GOLD: global initiative for chronic obstructive lung disease
FVC: forced vital capacity
Med: median
IQR: inter-quartile range
M: male
F: female
PaO_2_: arterial oxygen tension
PaCO_2_: arterial carbon dioxide tension
HCO_3_^−^: bicarbonate concentration
CRP: C-reactive protein
WBC: white blood cells
OR: odds ratio
CI: confidence interval
SMR: standardized mortality ratio
